# Primary Immune Response Provides Protective Efficacy against SARS-CoV-2 Reinfection

**DOI:** 10.31729/jnma.5538

**Published:** 2021-07-31

**Authors:** Rupendra Shrestha, Reena Shrestha, Ram Bahadur Khadka, Rabin Gyawali

**Affiliations:** 1 Department of Ophthalmology and Visual Sciences, Albert Einstein College of Medicine, New York, United States; 2 College of Medical Science and Teaching Hospital, Bharatpur, Chitwan, Nepal; 3 Department of Medical Laboratory Technology, Crimson College of Technology, Butwal, Nepal; 4 Department of Basic Sciences, Nepal Sanjivani Institute of Health Science, Dang, Nepal

**Keywords:** *COVID-19*, *immunity*, *reactivation*, *reinfection*

## Abstract

While there is absolutely no evidence to ensure recovered patients are either likely or unlikely to get reinfected. But studies in non-human primates indicate that reinfection of recovered patients is highly unlikely. It is also clear that primary immune responses or induced immunity to severe acute respiratory syndrome coronavirus 2 remain in circulation for several months and at least temporarily confer immunity to protect from reinfection. In addition, negative virus culture analysis of re-positive suggests that positive reverse transcriptase-polymerase chain reactions in recovered patients are more likely to be false-positive, or detection of genetic remnants of virus discharged from lesions of lungs or better sampling at the time of repeat analysis. However, emerging severe acute respiratory syndrome coronavirus 2 variants are likely to be causing the infections observed in some of the recovered patients.

## INTRODUCTION

Novel coronavirus disease 2019 (COVID-19) is an infectious respiratory disease caused by the emergence of severe acute respiratory syndrome coronavirus 2 (SARS-CoV-2), which poses a challenge to global public health. Yet, the immunity of this novel virus is not defined, and there is rising global speculation about the risk of reinfection. Few recent reports from china have confirmed the positive reverse transcriptase-polymerase chain reaction (RT-PCR) in numerous patients after complete recovery of clinical symptoms or with previous negative test results.^1-3^ Also, Korean CDC (Center for Disease Control and Prevention) reported similar findings that detected ribonucleic acid (RNA) in clinical samples of 447 recovered cases.^4^

## SARS-COV-2 REACTIVATION

Despite the fact that there is limited evidence, KCDC thus postulates the new hypothesis about the probability of COVID-1 9 viral reactivation instead of reinfection. Taking this into consideration, they issued testing guidelines that patients are only considered fully recovered if RT-PCR is tested negative twice within 24 hours.^5,6^ Conversely, another study indicated that repeat analysis of RT-PCR 48 hours apart is necessary to ensure virus clearance.^3^ Besides, laboratory tests, including D-dimer, absolute lymphocyte count, and antibody test in combination with RT-PCR negative testing, are essential to ensure that the virus is no longer transmitted by discharged or recovered patients.^3^ Few recent reports have shown that asymptomatic or moderately symptomatic or in close contact with patients recovered is also a means of transmission or infection in the population.^1,7,8^ Furthermore, persistent viral shedding has been recorded for an extended period of 60 days from the onset of symptoms, and even for 36 days after the recovery of symptoms.^9^ This suggests that patients with COVID-19 or those recovered may require prolonged isolation. Also, more precautions are obviously needed to be certain that a virus has not been reactivated after recovery, but the patient has fully recovered. Although reactivation may be rare or very unlikely, the possible risks are too high to ignore, warranting an urgent call for more in-depth research.

## PRIMARY IMMUNE RESPONSE AGAINST SARS-COV-2

**Figure 1:**
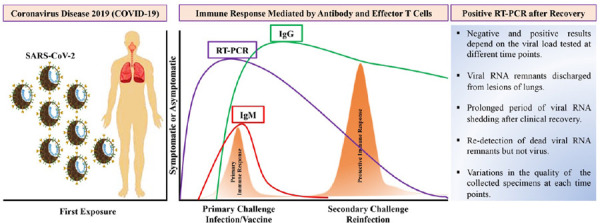
Schematic illustration of SARS-CoV-2 infection, an immune response to infection, variations of diagnostic tests, and a description of re-positive test after recovery.

Researchers from China performed experiments in rhesus macaques to investigate acquired immunity by re-exposing them with SARS-CoV-2 after confirmed recovery of initial infection. Interestingly, they found that reinfected monkeys didn't show signs of recurrence of infections, and virus replication was not detected in the nasopharyngeal swabs.^10^ A similar study from US groups demonstrated that 5log10 reductions in median viral load and neither led to respiratory failure or mortality.^11^ Another group developed six candidates deoxyribonucleic acid (DNA) vaccines formulated using variants of the critical viral protein. These investigational vaccines were immunized in adult rhesus macaques to investigate the immunological response against the virus. The study revealed that most of the vaccinated animals showed no detectable virus, while few showed dramatically lower viral loads compared to that of control group.^12^ Those studies on non-human primates (rhesus macaques) suggest that immunity (antibody- and cell-mediated immune responses) acquired following primary infection provided immunity against SARS-CoV-2 relapse (Figure 1).^10-12^

On exposure to SARS-CoV-2, the body triggers the primary immune response (antibody and effector T cells), which protects against infection (small peak-orange). Such immunity remains in the body, which provides protective efficacy against secondary challenges (large peak-orange). During the infection phase, RT-PCR may be used to detect viral nucleic acid in the early infection phase (blue line) followed by detection of IgM antibodies (red line), while IgG antibodies may be detected in the later infection phase which serves as a long-term immunity (green line).

It is therefore clear that higher antibody levels are directly proportional to lower levels of the virus that indicates neutralizing antibodies are a reliable marker of protection. Such findings raise the probability that immunological interventions will ultimately be effective for prevention and treatment. But it is critical to stress that notable variations exist between viral infection in macaques and humans. This suggests that rigorous clinical studies are required to evaluate immunity against SARS-CoV-2 reinfection in humans.

## RE-INFECTION ARE UNLIKELY AFTER RECOVERY

Besides, re-positive RT-PCR in recovered patients are unlikely to be caused by the reinfection from the residual virus, as described in Figure 1 . Actually, it emerges from the residual virus transferred to the upper respiratory tract (throat or nose) with a cough from the lower respiratory tract.^2^ RT-PCR will once again test absolutely negative upon full recovery of lesions in the lungs and clinical symptoms.^2^ Interestingly, KCDC has demonstrated negative results of virus isolation in cell culture on further analysis of respiratory samples from re-positive RT-PCR. Also, they showed that 96% positive for viral neutralizing antibodies in a serum sample of re-positive RT-PCR cases.^4^ In a row, experts in South Korea have confirmed that reactivation or reinfection is possibly due to the results of a false-positive test that is detected from the remains of dead viruses.^13^ According to new research, immunity to SARS-CoV-2 induced by infection or vaccination appears to be long-lasting. During an infection, cells that recognize the virus survive in the bone marrow and produce antibodies as needed.^14^ As a result, COVID-19 recovered individuals who were later immunized will not require boosters. While vaccinated people who have never been infected, or who have been infected but have not produced a strong immune response, will certainly need boosters. Vaccine breakthrough infection reported in some Nepalese might result from emerging variants circulating in Nepal. It is also possible that SARS-CoV-2 variants are less susceptible to vaccines than the wild-type virus.

Infection observed in some of the recovered patients is more likely as a result of SARS-CoV-2 emerging variants than reinfection with wild-type SARS-CoV-2. More research is required to assess if recovering patients are at risk of reinfection, and further precautions tend to be required to ensure that a patient has fully recovered from COVID-19 or is unable to reactivate or patients are fully vaccinated. Furthermore, genomic surveillance and vaccinations are only a key to drive evidence-based policies and implement effective control strategies in Nepal.

## References

[1] Lan L, Xu D, Ye G, Xia C, Wang S, Li Y (2020). Positive RT-PCR test results in patients recovered from COVID-19.. JAMA..

[2] Tao J, Hu Z, Liu J, Pang P, Fu G, Qian A (2020). Positive RT-PCR test results in discharged COVID-19 patients: reinfection or residual?. Research Square..

[3] Yuan J, Kou S, Liang Y, Zeng J, Pan Y, Liu L (2020). Polymerase chain reaction assays reverted to positive in 25 discharged patients with COVID-19.. Clin Infect Dis..

[4] Hoang T (2021). Systematic review and meta-analysis of factors associated with re-positive viral RNA after recovery from COVID-19.. J Med Virol..

[5] Smith J (2020). South Korea reports more recovered coronavirus patients testing positive again [Internet].. Reuters: Healthcare & Pharma.

[6] Alizargar J (2020). Risk of reactivation or reinfection of novel coronavirus (COVID-19).. J Formos Med Assoc..

[7] Ye F, Xu S, Rong Z, Xu R, Liu X, Deng P (2020). Delivery of infection from asymptomatic carriers of COVID-19 in a familial cluster.. Int J Infect Dis..

[8] Huang L, Zhang X, Zhang X, Wei Z, Zhang L, Xu J (2020). Rapid asymptomatic transmission of COVID-19 during the incubation period demonstrating strong infectivity in a cluster of youngsters aged 16-23 years outside Wuhan and characteristics of young patients with COVID-19: A prospective contact-tracing study.. J Infect..

[9] Li J, Zhang L, Liu B, Song D (2020). Case Report: viral shedding for 60 days in a woman with COVID-19.. Am J Trop Med Hyg..

[10] Deng W, Bao L, Liu J, Xiao C, Gao H, Xue J (2020). Reinfection could not occur in SARS-CoV-2 infected rhesus macaques.. Science..

[11] Chandrashekar A, Liu J, Martinot AJ, McMahan K, Mercado NB, Peter L (2020). SARS-CoV-2 infection protects against rechallenge in rhesus macaques.. Science..

[12] Yu J, Tostanoski LH, Peter L, Mercado NB, McMahan K, Mahrokhian SH (2020). DNA vaccine protection against SARS-CoV-2 in rhesus macaques.. Science..

[13] France N (2020). Not COVID-19 reactivation, just false positive tests confirm South Korean experts [Internet]..

[14] Turner JS, Kim W, Kalaidina E, Goss CW, Rauseo AM, Schmitz AJ (2021). SARS-CoV-2 infection induces long-lived bone marrow plasma cells in humans.. Nature..

